# Supplementary material for: Hydroxypropyl-β-Cyclodextrin Depletes Membrane Cholesterol and Inhibits SARS-CoV-2 Entry into HEK293T-ACEhi Cells
Source: Pathogens. 2023 Apr 27;12(5):647. doi: 10.3390/pathogens12050647 (PMC10224288; doi:10.3390/pathogens12050647)
Supplement: Supplementary file 1 [file pathogens-12-00647-s001.zip › pathogens-2312568 supplementary.pdf]

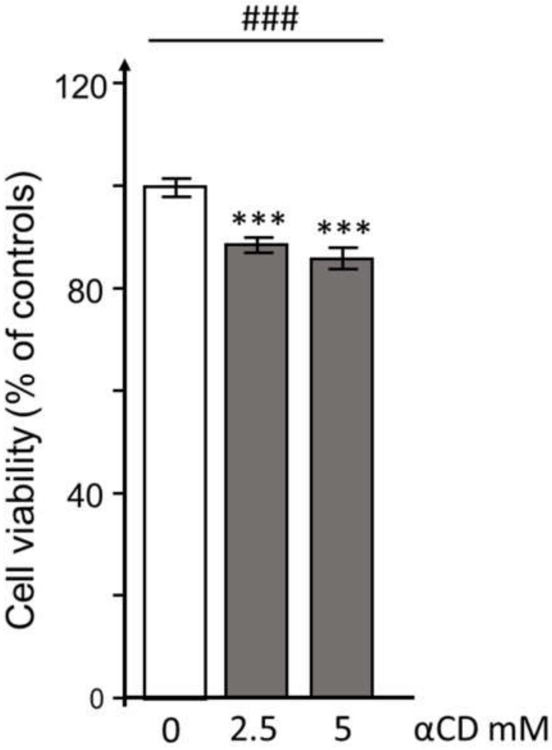

Figure S1. *α*-cyclodextrin induces cytotoxic effect in HEK293T-ACEhi cells. HEK293T-ACEhi cells were treated overnight with 2.5 or 5 mM CD or vehicle (controls) and then tested for cell viability (MTT test). *α*CD exposure results cytotoxic at concentrations at which HPβCD was not. Data are the mean ± SEM ($n = 8$). The concentration effect of *α*CD on cell viability was determined with a one-way ANOVA (### $p < 0.001$) followed by post-hoc Dunnett's test vs. controls (*** $p < 0.001$). *α*CD: alpha-cyclodextrin.

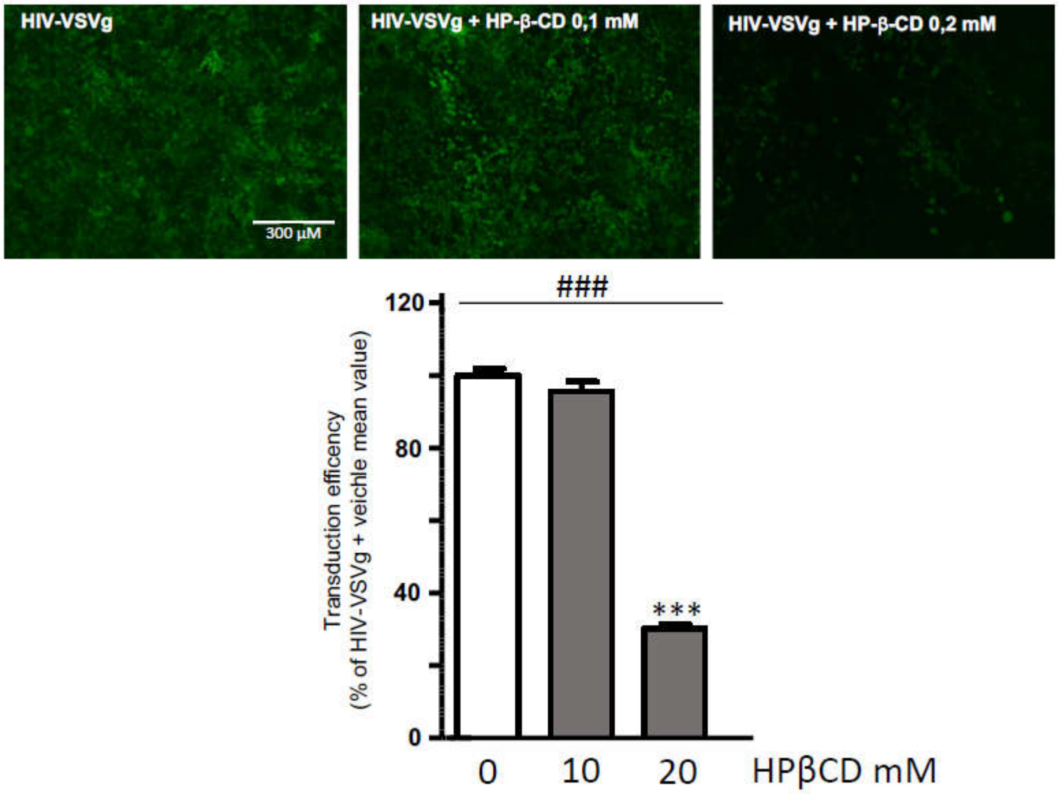

Figure S2. A 20 mM dose of hydroxypropyl-β-cyclodextrin (HPβCD) reduces entry of lentiviral particles pseudotyped with VSVg Envelope glycoprotein into HEK293T-ACEhi cells. HEK293TACEhi cells were incubated overnight with pseudotyped viral particles containing a GFP expression cassette in the presence of 10 or 20 mM of HPβCD or vehicle. Three days post-transduction GFP expression qualitative analysis was performed by EVOS Cell Imaging Systems Panel on live-HEK293T-ACEhi cells (see upper panels) before proceeding to the quantitative analysis by flow cytometry (lower panel). Scale bar = 300 μm. Data are expressed as mean ± SEM (n = 12, four independent experiments). The one-way ANOVA (### $p < 0.001$) followed by the post-hoc Dunnett's test (*** $p < 0.001$ vs. vehicle treated cells) was used to analyze the differences between the experimental conditions.
